# [More] evidence to support oral health promotion services targeted to smokers calling tobacco quitlines in the United States

**DOI:** 10.1186/1471-2458-13-336

**Published:** 2013-04-11

**Authors:** Jennifer B McClure, Karin Riggs, Jackie St John, Sheryl L Catz

**Affiliations:** 1 Group Health Research Institute, Seattle, WA, USA

**Keywords:** Oral health promotion, Smoking, Tobacco, Oral disease

## Abstract

**Background:**

Prior research demonstrated a need and opportunity to target smokers calling a free, state-funded tobacco quitline to provide behavioral counseling for oral health promotion; however, it is unclear whether these results generalize to tobacco quitline callers of higher socioeconomic status receiving services through commercially-funded quitlines. This knowledge will inform planning for a future public oral health promotion program targeted to tobacco quitline callers.

**Methods:**

We surveyed smokers (n = 455) who had recently received tobacco quitline services through their medical insurance. Participants were asked about their self-reported oral health indicators, key behavioral risk factors for oral disease, motivation for changing their oral self-care behavior, and interest in future oral health promotion services. Where applicable, results were compared against those from a representative sample of callers to a free, state-funded quitline (n = 816) in the same geographic region.

**Results:**

Callers to a commercially-funded quitline had higher socioeconomic status, were more likely to have dental insurance, and reported better overall oral health indicators and routine self-care (oral hygiene, dental visits) than callers to a state-funded quitline. Nevertheless opportunities for oral health promotion were identified. Nearly 80% of commercial quitline callers failed to meet basic daily hygiene recommendations, 32.8% had not visited the dentist in more than a year, and 63.3% reported daily alcohol consumption (which reacts synergistically with tobacco to increase oral cancer risk). Nearly half (44%) were interested in learning how to improve their oral health status and, on average, moderately high levels of motivation for oral health care were reported. Many participants also had dental insurance, eliminating an important barrier to professional dental care.

**Conclusions:**

Future public oral health promotion efforts should focus on callers to both free state-supported and commercially-funded tobacco quitlines. While differences exist between these populations, both groups report behavioral risk factors for oral disease which represent important targets for intervention.

## Background

Oral disease is a significant public health threat which affects millions of people. It includes common oral diseases such as periodontitis and dental caries, and less common oral and pharyngeal cancers. Together these diseases exact a large human toll. Oral disease not only results in unnecessary pain, potential physical disfigurement, and emotional suffering, it puts individuals at greater risk for subsequent morbidity and mortality [1]. For example, periodontal oral infections are associated with increased risk of cardiovascular disease, stroke, adverse pregnancy outcomes, diabetes, obesity, and non-oral cancers [1-5]. Treating acute disease and preventing the onset of future oral disease are important public health goals.

Tobacco users are at particularly high risk for oral disease compared to people who do not use tobacco [1,6]. Unfortunately, many smokers fail to seek routine professional dental care [6-8]. As a result, additional public oral health efforts may be needed to reach smokers and to augment the preventive care provided by dental practitioners. Routine oral hygiene such as daily brushing and flossing, use of fluoride products, and lifestyle changes such as reducing tobacco and alcohol use are critical to preventing disease [1]. In fact, encouraging proper self-care at home is now an accepted practice of preventive intervention [9] and can be influenced by behavioral counseling [10,11], prompting a recent recommendation that public health officials consider leveraging the existing tobacco quitline infrastructure in the United States to counsel tobacco users about both smoking cessation *and* better oral health care [8].

Tobacco quitlines provide motivational and cognitive behavioral counseling for tobacco cessation, primarily through proactive counseling calls (i.e., calls initiated by the quitline based on a pre-determined call schedule established after one enrolls in the services). Participants typically receive supplemental mailed materials and may also have access to online intervention materials or even text-based messaging support. Tobacco quitlines are ubiquitous in North America. At present, all 50 US states, the District of Washington, Puerto Rico, Guam, and 10 Canadian provinces offer free tobacco quitline programs. Other smokers receive services from the hundreds of commercially-funded quitline programs supported through health insurers, employers, and other private funders. According to data from the North American Quitline Consortium (NAQC), in 2009 alone more than 380,000 tobacco users received services from their quitline members [12]. The Global Quitline Network includes additional service providers in Europe and Asia. Expanding oral health promotion activities provided through the existing quitline infrastructure could reach a sizable population, nationally and internationally, and be an innovative way to reach individuals who are at particularly high risk for oral disease. It would also take advantage of an existing cadre of trained behavior change professionals, and the preventive care counseling offered could augment the standard care provided by dental care providers and even encourage utilization of their services. That is, in addition to receiving tobacco cessation counseling, quitline callers could be informed about the risks of oral disease and counseled on ways to improve their oral health through lifestyle risk factor modification (e.g., quitting smoking, limiting alcohol use, proper nutrition, fluoride use, brushing and flossing daily, and seeing a dentist regularly).

To assess the feasibility of this strategy in the U.S., we previously surveyed a representative sample of callers to the Washington State Quitline (WAQL; n = 816) [8]. WAQL provides free tobacco cessation counseling to all eligible callers. Persons are typically eligible if they do not have access to nicotine dependence treatment services through other means (e.g., coverage provided by an employer or through private health insurance) or if they are in a high risk priority group, such as pregnant smokers. The results of this survey demonstrated an important opportunity to promote better oral health among tobacco quitline callers by intervening to prevent future oral disease. Among tobacco users who still had their natural teeth (79.3% of those surveyed), most failed to meet the American Dental Association’s (ADA) recommendations for daily brushing and flossing (83.9%) and most had not visited the dentist in more than a year (52.6%). Although, relatively few people in this sample self-reported a diagnosis of gum disease (21.6%) or dental bone loss (20.4%). If these reports are accurate, it underscores the opportunity for preventative care, to reduce the risk of future disease. If, however, these rates are an underestimation of oral disease in this population, it speaks to the opportunity to better educate smokers about the signs of oral disease and need for regular professional dental screenings. We also found that many tobacco users were open to learning more about ways to improve their oral health (57.4%), were receptive to be counseled by their quitline coach (48.2%), or were open to receiving oral health intervention materials by mail (62.7%) or Internet (50.0%), so there is evidence that many smokers contacted through the quitlines are receptive to oral health promotion efforts.

These data are encouraging, but it is unclear whether the results generalize to smokers recruited through commercially-funded quitlines. In contrast to state-funded quitlines, which reach smokers of lower socio-economic status and are often limited to persons with no private health insurance, commercial quitlines are typically funded by health plans or employers. As such, they reach tobacco users who are of higher socioeconomic status and, therefore, may have different oral health needs, interests and intervention opportunities. The current paper examines self-report oral health indicators, behavioral oral health risk factors, motivation for behavior change, and interest in oral health promotion among medically-insured smokers receiving tobacco quitline services. We sought to replicate the outcomes previously reported among state quitline participants and to extend them by examining additional indicators of oral disease, behavioral risk behaviors, and motivation for change. These findings not only enhance our understanding of smokers’ oral health needs, but will help inform future efforts to design oral health promotion programs that can be integrated into the standard counseling offered to smokers through tobacco quitlines.

## Methods

### Participants

Callers were surveyed from a commercially-funded quitline offered to members of a regional U.S. health plan through their medical insurance (n = 455). All participants lived in Washington state and were insured by Group Health Cooperative. Tobacco users who consecutively enrolled in the quitline program between July and September 2011 were identified using health plan enrollment files and quitline intake data. Enrollees who were adult smokers at the time of intake and had reported that they could read and write in English (n = 829) were each mailed a written survey and a $2 pre-incentive in February 2012. Invitees who failed to return their survey within several weeks after the first mailing received a second reminder mailing. Fifty-five percent of those invited returned surveys and received $20 as a thank you. One non-responder was learned to be deceased. All data collection was conducted by the Group Health Research Institute. All study activities were approved by the Group Health Institutional Review Board.

### Assessment measures and analyses

Assessment measures included standardized items from the National Health and Nutrition Examination Survey (NHANES) and the Center for Disease Control’s Behavioral Risk Factor Surveillance System Survey (BRFSS). Items included socio-demographic variables, dental insurance status (yes/no), lifetime smoking and current tobacco use (smoked even a puff in the past 7 days [yes/no], dentate status (whether or not respondents had at least some permanent natural teeth [yes/no]), self-reported health of their teeth and gums (ranging from ‘poor’ to ‘excellent’ on a 5 point Likert scale), oral health status (number of permanent teeth removed due to disease, perceived gum disease [yes/no], self-rated overall health of teeth and gums [ranging from ‘poor’ to ‘excellent’], prior treatment for gum disease [yes/no], prior diagnosis of dental bone loss [yes/no], and oral hygiene behaviors (times per day brush teeth, times per day use an interdental cleaning device, times per week floss). All items are available in the public domain. For greater clarity, one NHANES item was modified slightly to read “In the last seven days, how many days did you use dental floss or any device other than a toothbrush to clean between your teeth?” Time since last visit to a dentist or dental clinic and time since last dental cleaning were also assessed using standardized BRFSS items. Alcohol use was assessed with the AUDIT-C, a well-validated three-item brief screening of drinking frequency and quantity [13,14]. Participants were also asked about their interest in learning more about how to improve the health of their teeth and gums [yes/no], speaking with a tobacco quitline coach about ways to improve their oral health [yes/no], and interest in receiving oral health information via Internet or mail about ways to reduce their oral health disease [yes/no]. Participants also indicated their motivation for taking good care of their teeth and gums, seeing a dentist in the next 6 months, daily brushing, daily flossing, and brushing their tongue daily. Each of these items was rated on a 7 point Likert scale from ‘not at all’ to ‘very’ motivated.

Consistent with current ADA recommendations, persons were judged to be meeting oral hygiene recommendations if they reported flossing each day in the last week and brushing at least twice a day. Per convention, current smokers were defined by a lifetime history of smoking at least 100 cigarettes and smoking, even a puff, in the past 7 days. Persons reporting any natural teeth were considered dentate. Persons reporting loss of all permanent, natural teeth were considered edentulous [15].

Data from the current survey were summarized using descriptive statistics. Where applicable, outcomes were compared with data previously collected from callers to a state-funded quitline using chi-square for categorical measures and t-tests for continuous measures. To prevent duplicative data reporting, only effect sizes and *p* values determined using logistic regression are presented to indicate the significance of these comparisons; previously published descriptive statistics from the state-funded quitline sample are not presented.

## Results

### Participant characteristics

Participant characteristics are presented in Table 1. Respondents were predominantly non-Hispanic white, middle-aged, females. The majority had at least some college education and two-thirds were employed. More than half had a household income exceeding $40,000/year and three-quarters reported dental insurance coverage.

**Table 1:** Participant characteristics

	**All participants**	**Dentate participants**
	**n = 455**	**n = 384**
	**Mean (SD)**	**Mean (SD)**
Age	48.3 (13.8)	46.7 (13.9)
		
	**N (%)**	**N (%)**
Female	296 (65.1)	247 (64.3)
White, non-hispanic	384 (84.4)	322 (83.9)
Married or living as married	269 (59.1)	226 (58.9)
Education		
High school or less education	152 (33.4)	125 (32.6)
Some college	202 (44.4)	166 (43.2)
College or post-graduate degree	101 (22.2)	93 (24.2)
Employed	298 (65.5)	262 (68.2)
Household income ^a^		
< $20,000	69 (15.2)	56 (14.6)
$20-39,999	115 (25.3)	93 (24.2)
$40,000 – 59,000	77 (16.9)	64 (16.7)
≥ $60,000	153 (33.6)	135 (35.1)
Dental insurance (private or government funded)	358 (78.7)	309 (80.5)
Tobacco use		
Cigarette smoker ^b^	274 (60.2)	233 (60.7)
Pipe, cigar, or bidis	9 (2.0)	9 (2.3)
Oral tobacco	9 (2.0)	7 (1.8)
Alcohol user	329 (74.3)	263 (76.4)

^a^ Percents do not total 100%. Some respondents (7%) preferred not to answer.

^b^ Defined as smoking 100 cigarettes in lifetime and smoked in past 7 days.

### Oral health indicators

Oral health indicators are presented in Table 2. Eighty-five percent of respondents had some or all of their natural teeth. Nearly half of these had lost one or more teeth due to disease. Approximately 15% of the total sample had lost all of their natural teeth. One third of dentate respondents reported prior treatment for gum disease, such as scaling or root planing, and a quarter indicated they had been diagnosed with dental bone loss. Based on their current symptoms, 30% believed they may have gum disease.

**Table 2:** Self-report oral health indicators

	**All participants**	**Dentate participants**
	**n = 455**	**n = 384**
	**N (%)**	**N (%)**
Edentulous (lost all natural, permanent teeth)^a,b^	68 (14.9)	n/a
Self-rated health of teeth & gums as ‘good’ or better ^a^	272 (59.8)	271 (70.6)
Believe may have gum disease based on current symptoms (e.g., swollen, receding, sore, or infected gums or loose teeth)^a^	116 (25.5)	116 (30.2)
One or more permanent teeth removed due to Disease^c^	231 (50.7)	175 (45.7)
Ever received treatment for gum disease (such as scaling or root planing)^a^	136 (29.9)	136 (35.4)
Ever had teeth become loose without injury	73 (16.0)	73 (19.0)
Ever diagnosed with dental bone loss^a^	97 (21.3)	96 (25.0)
Ever diagnosed with oral cancer	4 (0.9)	2 (0.5)

^a^ Source: 2009–2010 NHANES.

^b^ Dentate status was not available for 3 participants.

^c^ Source: 2008 BRFSS, oral health module.

### Oral health self-care

Self-reported oral health self-care behaviors among dentate respondents are presented in Table 3. Two thirds of dentate respondents met the ADA recommendation to brush their teeth daily, but only 27.6% reported daily flossing. The median number of times people flossed per week was 3, as was the median number of days per week they flossed. In total, only 85 people (22.1%) met ADA recommendations for both daily brushing and flossing; 77.9% failed to meet this basic daily hygiene recommendation.

**Table 3:** Oral health behaviors among dentate respondents

	**Dentate participants**
	**n = 384**
	**N (%)**
Met ADA recommendation: brush twice daily	252 (65.6)
Met ADA recommendation: floss 7 days week	106 (27.6)
Met ADA recommendation: brush twice and floss daily	85 (22.1)
Visited dentist or dental clinic in last 12 months^a^	258 (67.2)
Had teeth cleaned in last 12 months^b^	242 (63.0)

^a^Source: 2009–2010 NHANES.

^b^Source: 2008 BRFSS, oral health module.

In terms of professional dental care, approximately two-thirds had their teeth cleaned in the past 12 months and a similar proportion had visited a dentist in the past year.

### Other oral health risk behaviors

The oral health survey was conducted up to 8 months after smokers had enrolled in the tobacco quitline program. By the time of this assessment, approximately 40% reported no longer smoking, but most continued to smoke and a small number reported use of other forms of tobacco products (see Table 1). The majority of respondents also reported alcohol use (74.3%) and 63.3% (n = 288) reported daily alcohol consumption. Thirty-four percent of women (n = 100) and 40.3% percent of men (n = 64) had AUDIT-C scores indicative of problem drinking (i.e., scores ≥ 3 for women and 4 for men).

### Interest in oral health promotion

Overall, respondents had moderate to high levels of motivation to take good care of their oral health. Motivation levels by specific oral health self-care behaviors of interest are reported in Table 4.

**Table 4:** **Motivation for oral health care**^**a**^

	**All participants**	**Dentate participants**	**Dentate participants not meeting ADA oral hygiene recommendations **^**b**^
	**n = 455**	**n = 384**	**n = 288**
	**Mean (SD)**	**Mean (SD)**	**Mean (SD)**
Take good care of my teeth and gums	5.7 (1.4)	5.8 (1.2)	5.6 (1.2)
Floss my teeth daily	5.2 (2.0)	5.5 (1.7)	5.2 (1.8)
Brush my tongue daily	5.9 (1.7)	5.9 (1.7)	5.8 (1.7)
Brush my teeth daily	6.4 (1.4)	6.6 (1.0)	6.5 (1.1)
See a dentist in the next 6 months	5.8 (1.8)	6.1 (1.5)	6.0 (1.5)

^a^ Each item measured on a 7 point Likert scale: 1 = not at all motivated, 4 = somewhat motivated, and 7 = very motivated.

^b^ Defined as brushing twice a day and flossing daily.

Moderate interest in future oral health promotion intervention was also observed. Approximately half (n = 198, 43.5%) of all respondents said they were interested in learning more about ways to improve their oral health, with many interested in receiving this information either over the Internet (n = 223, 49.0%) or by mail (n = 213, 46.8%). Fewer were interested in talking with a quit coach about ways to improve their oral health (n = 132, 29.0%), but this may reflect the fact that many participants had completed their quitline counseling program at the time of the survey.

Interest in oral health promotion differed between dentate and edentulate smokers. More dentate smokers were interested in learning how to improve their oral health (47.0% vs. 28.8%, p = .006) and interested in receiving materials by Internet (51.6% vs. 38.8%, p = .054), but fewer dentate respondents were interested in receiving oral health materials by mail (45.2% vs. 59.7%, p = .029). There was no difference in respondents’ interest in talking with a quit coach about their oral health (29.4% [dentate] vs. 29.9% [edentulate], p = .94).

### Comparison with state-funded quitline population

Compared to smokers sampled from a state-funded quitline [8], participants were more likely to be non-Hispanic white (OR = 1.36, 95% CI 1.00 – 1.85; p < .05), to have some college education (OR = 2.12, 95% CI 1.67-2.69; p < 0.001), to have annual household incomes at or above $40,000 (OR = 6.23, 95% CI 4.81-8.06; p < 0.001), and to have dental coverage (OR = 5.94, 95% CI 4.49-7.86; p <0.001). Differences in oral health indicators were also observed. Fewer participants in the current sample had lost all of their natural teeth (OR = 0.70, 95% CI 0.52-0.96; p = .03) and fewer thought they could have gum disease based on their symptoms (OR = 0.58 95% CI 0.44-0.76; p < 0.001), but more reported they had been treated for gum disease in the past (1.62, 95% CI 1.24-2.11; p <0.001). A similar proportion of each group had been diagnosed with oral cancer (0.9% vs. 1.0%, OR = 0.89, 95% CI 0.38-2.10; p = .97).

Finally, differences in oral health behaviors, motivation and interest in oral health promotion services were also observed. A greater proportion of commercial quitline callers met ADA recommendations for brushing (OR = 1.4 95% CI 1.09-1.86; p = .01), flossing (OR = 1.53 95% CI 1.14-2.06; p = 0.005), or daily oral hygiene (combined brushing and flossing) (OR = 1.52 95% CI 1.10-2.10; p = .01); and 20% more participants had visited the dentist last year (OR = 1.96, 95% CI 1.53-2.51; p < .001). Commercial quitline participants were also more motivated to see a dentist (mean score 5.8 vs. 5.3, p < .001) and floss daily (mean score 5.4 vs. 5.0, p = .01), but no more motivated to brush daily (6.3 vs. 6.4, p = .42). They were also less interested in oral health promotion services than were callers to the state quitline program, defined as interest in learning how to improve their oral health (OR = 0.57, 95% CI 0.45-0.72; p < .001), interest in talking with a quitline counselor (OR = 0.44, 95% CI 0.35-0.56; p < .001), or interest in receiving oral health promotion information by mail or Internet (OR = 0.68, 95% CI 0.52-0.88; p = 0.003).

## Discussion

While clear differences exist between smokers seeking services through a commercially-funded versus a state-funded tobacco quitline, the survey results indicate that opportunities to promote better oral health do exist among more economically-advantaged smokers calling a tobacco quitline. For one, most smokers still had some or all of their natural teeth, making preventative care and lifestyle risk factor modification important. Additionally, most smokers had dental coverage (85.1%). This was a significant potential barrier to care among state quitline callers, of whom only 42.4% reported dental insurance, including those covered through Medicaid services which have since been eliminated in Washington. That most of the commercial quitline callers have dental insurance increases the possibility that they could follow through on recommendations for routine professional dental care. Two-thirds of participants had visited a dentist in the past year, but one third had not. This proportion is lower than that observed in the general population residing in the same geographic region (76.0%), based on data from the 2010 Washington state BRFSS [16]. Educating smokers about the importance of routine dental care and providing referrals for treatment could boost uptake rates. Persons who do not have insurance could be referred to more affordable treatment options, such as care provided through local dental schools.

Even in the absence of professional dental care, opportunities exist to improve smokers’ oral hygiene and lifestyle risk factors. Nearly 80% of those surveyed failed to meet basic daily hygiene recommendations (brushing and flossing) and more than 63% reported daily alcohol consumption. Each of these behaviors place smokers at elevated risk for future periodontal disease, cavities, and oral cancer [17-19] and represent important targets for intervention. In fact, a dual counseling focus on smoking cessation and oral health promotion that addresses these behaviors could have a positive synergistic effect on both tobacco cessation *and* improved oral health outcomes. This focus could be integrated into a standard cognitive behavioral intervention for nicotine dependence treatment. It is not uncommon to advise smokers preparing to quit to have their teeth cleaned (i.e., to remove tobacco stains and start fresh), to brush their teeth or floss as a way of coping with urges to smoke after quitting, or to limit/avoid alcohol use since it interferes with one’s ability to stop using tobacco. This advice could be extended to include discussion of the interconnection between one’s oral health and their overall health, the importance of proper daily hygiene and use of fluoride products, and the synergistic effects of tobacco and alcohol on oral cancer risk [18]. Smokers can also be encouraged to chew sugar-free gum. Chewing gum is another common strategy for coping with cravings to smoke, and sugar-free gum can reduce plaque and decrease one’s risk of dental caries [20-22].

A combined oral health promotion-tobacco cessation program would also be responsive to the U.S. Surgeon General’s national call to action to promote oral health [23]. Our data indicate many smokers would be receptive to this intervention. Most smokers in the current survey endorsed moderately high levels of motivation for taking good care of their teeth and gums and nearly half said they were interesting in learning more about how to improve their oral health. In fact, since many elements of this intervention could be interwoven into a standard smoking cessation program without delineating it as a separate intervention, all smokers could receive this integrated intervention. These efforts could never replace the important preventative and acute care provided by dental health professionals, but this expanded health focus could be an innovative way to augment this care and better meet the multi-factorial health needs of smokers.

Clearly, future research must confirm the effectiveness of the proposed intervention strategy, but data collected from two separate representative tobacco quitline populations now support the need and opportunity to pursue this effort in conjunction with both publically and commercially funded tobacco quitline programs.

The current survey is strengthened by inclusion of a large group of consecutively sampled quitline participants and our ability to compare outcomes across diverse quitline populations. Because all participants were from Washington state, it is possible that the results may not generalize to smokers in other geographic areas, but we are unaware of any reason that callers in Washington would have systematically worse oral health self-care behaviors than smokers in other states. Furthermore, the data suggest that participants’ self-reported oral health indicators (i.e., edentulism, self-rated oral health) are similar to those reported among smokers in other surveys [6,15], providing more confidence in the generalizability of these results.

## Conclusions

Many dentists commonly counsel their patients about quitting smoking. Our data suggest an opportunity for the reverse—for smoking cessation counselors to encourage better oral health care. This strategy alone will not address the oral health needs of all smokers in the U.S (an estimated one in five adults) [24], but it could represent a creative strategy for reaching an important segment of this population—smokers ready to quit smoking and ready to take action to improve their health. This research provides further evidence of the opportunity for an integrated oral health promotion-tobacco cessation intervention offered through quitlines and illustrates important behavioral targets for oral health promotion among smokers. Further evaluation of this proposed intervention strategy is warranted.

## Abbreviations

ADA: American Dental Association; BRFSS: Behavioral Risk Factor Surveillance System; GHRI: Group Health Research Institute; NHANES: National Health and Nutrition Examination Survey; NAQC: North American Quitline Consortium; SES: Socio-economic status; WAQL: Washington Quitline.

## Competing interests

The authors declare that they have no competing interests.

## Authors’ contributions

JBM is responsible for developing the research concept, securing research funding, drafting the survey, interpreting the results and drafting the manuscript. KR assisted in drafting the survey content, analyzing the data, and preparing the manuscript. JSJ provided day-to-day project oversight at GHRI, assisted in the survey design, and provided comments on the manuscript. SLC assisted in data interpretation and preparation of the manuscript. All authors read and approved the final manuscript.

## Pre-publication history

The pre-publication history for this paper can be accessed here:

http://www.biomedcentral.com/1471-2458/13/336/prepub
